# Is There a Higher Frequency of Postoperative Depression in Patients Undergoing Awake Craniotomy for Brain Tumors?: A Prospective Study

**DOI:** 10.7759/cureus.19877

**Published:** 2021-11-24

**Authors:** Saqib Kamran Bakhshi, Anum Sadruddin Pidani, Mujtaba Khalil, Muhammad Shahzad Shamim

**Affiliations:** 1 Neurosurgery, Aga Khan University Hospital, Karachi, PAK; 2 Community Health Sciences, Aga Khan University Hospital, Karachi, PAK; 3 Research, Aga Khan University Hospital, Karachi, PAK

**Keywords:** depression, psychological stress, fear, brain tumor, awake craniotomy

## Abstract

Introduction

Brain tumor resection under awake settings may cause significant psychological stress, which may lead to perioperative anxiety and depression. We conducted a prospective study to compare postoperative depression in patients undergoing awake craniotomy (AC) for tumor resection and compare it with patients undergoing tumor resection under general anesthesia (GA).

Methods

We conducted a prospective study at a tertiary care hospital. Non-probability consecutive sampling was performed, and patients with a preoperative diagnosis of depression or with any other medical comorbidities that could precipitate depression were excluded. Two separate questionnaires, the Patient Health Questionnaire-9 (PHQ-9) Scale and the Karnofsky Performance Score (KPS), were used to screen depression and assess functional status, respectively.

Results

Ninety-six patients met the inclusion criteria and were included in the study. Out of these, 37 (38.1%) had undergone awake craniotomy and 59 (60.8%) had undergone conventional craniotomy (CC) (under general anesthesia) for brain tumor resection. To standardize our method, we ensured that the demographic variables, including mean age, gender, educational status, marital status, and socioeconomic conditions, were comparable between both groups. Postoperative functional status fared better in patients who underwent awake craniotomy (p = 0.03). The total number of patients suffering from postoperative clinical depression, according to the PHQ-9 Scale, was 41 (42.7%), of which 12 (12.5%) were in the awake craniotomy group and 29 (30.2%) were in the conventional craniotomy group. The median PHQ-9 Scale score in the awake craniotomy group was 6 (range: 3-10), which was less than the median score in the conventional craniotomy group, which was 9 (range: 4-12). This difference, however, was not statistically significant (p = 0.06).

Conclusion

Resection of brain tumors under awake conditions is not likely associated with any additional incidence of postoperative depression when compared with resection of tumors under general anesthesia.

## Introduction

The World Health Organization defines depression as “loss of pleasure in everyday life chores and loss of self-esteem.” It is characterized by persistent sadness and a lack of interest or pleasure in previously enjoyable activities. Moreover, it is estimated that more than 350 million people, or up to 5% of the world’s population, suffer from depression [1,2]. The incidence is higher in patients diagnosed with systemic malignancies and brain tumors, with some studies citing that it may be present in up to 40% of patients [3,4]. Awake craniotomy (AC) for resection of eloquent area brain tumors is becoming the norm of care across the world, which involves intraoperative brain mapping, neuro-navigation, and real-time monitoring of the patients’ neurological function while they are awake, thus minimizing risks and improving outcomes [5-7]. Other significant advantages of AC include shorter operating room times, early postoperative recoveries, and shorter stays in the hospital [8].

Several authors have addressed the prevalence of depression and the resulting impairment in the quality of life in patients with brain tumors [9,10]. There is, however, insufficient literature on the demerits of undergoing an AC with regard to mental health [11,12]. It is evident that patients with brain tumors are already at a higher risk of mental health disorders, specifically anxiety and depression. Adding to that, there is more concern for patients undergoing longer cranial surgery while being awake, as it may further affect patients’ mental health, increasing the likelihood of a post-traumatic stress disorder [11,12].

A few authors have looked at common mental disorders in patients undergoing AC. Ruis et al. conducted a prospective study on preoperative anxiety in patients who underwent AC for brain tumors and reported depression in 25% of the patients, the majority of whom were females [11]. Potters et al. concluded that preoperative anxiety and fear can impair intraoperative assessment and can lead to a higher incidence of post-traumatic stress disorder after AC [12]. Hejrati et al. reviewed their experience with 20 patients undergoing AC and did not find a significant difference in postoperative depression [13]. However, they did report that preoperative anxiety significantly increased the feeling of postoperative pain and hindered daily activity up to postoperative day 3 [13]. Although AC are increasingly being performed and reported, there is still a dearth of literature and understanding regarding its psychological implications on patients, particularly in a low- and middle-income country such as Pakistan. The objective of this study was to compare the incidence of postoperative anxiety and depression in patients undergoing AC for brain tumors and those undergoing conventional craniotomy (CC) under general anesthesia (GA).

## Materials and methods

A prospective cross-sectional study was conducted at a tertiary care setting in Karachi, Pakistan. Our study included 96 patients with a confirmed diagnosis of primary supratentorial brain tumors at variable stages. These patients were approached in neurosurgery wards, neurosurgery and oncology outpatient clinics, and oncology day-care suites from November 2017 to July 2018. Non-probability consecutive sampling was done, and all patients who met the eligibility criteria of the study and consented to participate were included.

The inclusion criteria included all adult patients (aged 18 years and above) who had undergone craniotomy for excision of brain tumors at our hospital and were in variable stages of postoperative treatment, such as chemotherapy, radiotherapy, or surveillance. Informed consent was taken from all participants. Patients who had a confirmed diagnosis of depression for about one year prior to the diagnosis of a brain tumor, those who were confused or incoherent with impaired speech, those with coexisting systemic malignancies, or those having any severe medical illness such as liver cirrhosis, hepatitis, or nephropathy that can alter mental status were excluded from the study.

Medical records were reviewed to determine the participants’ eligibility, and potentially eligible participants were approached by the authors during a scheduled follow-up visit at neurosurgery and oncology outpatient clinics and inpatient hospital stay post-surgery. Each patient, after the consent, was interviewed for 15-20 minutes to complete a structured pretested questionnaire for assessing predictor variables and Patient Health Questionnaire-9 (PHQ-9) Scale for the screening of depression [4]. The questionnaire was also pilot tested on 10 participants before the actual administration.

We divided all associated factors into three distinct categories, namely, patient-related, tumor-related, and treatment-related categories; the latter two were assessed using medical record review. Patient-related factors comprised demographic and socioeconomic variables including age, gender, marital status, number of dependents, children under 18 years, education, occupation, employment status, residency, traveling cost, caregiver support, current smoking status, past/current medical illness, history of psychological distress, strategies to handle stress (including but are not limited to isolation, aggression, prayers, crying, sleeping, addiction, and mind diversions), and functional status. Each participant’s functional status was assessed using the Karnofsky Performance Score (KPS); scores of less than 70 were indicative of impaired functional status. Moreover, socioeconomic status was computed using factorial analysis.

Tumor-related factors included tumor histology, tumor grade, recurrence, hemispheric lateralization, first symptoms, brain structures involved, and cognitive impairment. Furthermore, treatment-related factors included stage of treatment, number of chemotherapy cycles, duration since diagnosis, radiation therapy, current use of steroids and antiepileptic drugs, and treatment cost.

Statistical analysis was done using the Statistical Package for Social Sciences (SPSS Inc.) version 22. Means and standard deviation were calculated for continuous data with normal distribution, whereas median and interquartile range (IQR) were calculated for continuous data with skewed distribution. Percentages and proportions were calculated for categorical data. Chi-square test and T-test were used to compare categorical variables and means, respectively. A P-value of less than 0.05 was considered significant.

## Results

Our study included 96 participants, of which 37 (38.5%) had undergone awake craniotomy (AC) and 59 (61.4%) had undergone conventional craniotomy (CC) under general anesthesia. The basic demographics including mean age, gender distribution, education level, and socioeconomic status were comparable between the two groups. The details of all these factors are mentioned in Table 1.

**Table 1:** Comparison of basic demographics between the two groups SES: socioeconomic status

Parameters	Awake craniotomy ( N = 37)	Conventional craniotomy ( N = 59)	P-value
Age (years)	Mean: 41.56 ± 12.28	Mean: 43.72 ± 11.71	0.39
Gender			
Male	21 (56.76%)	42 (71.19%)	0.18
Female	16 (43.24%)	17 (28.81%)	
Educational status			
Class 1–9	6 (16.22%)	13 (22.03%)	0.81
Matriculation	6 (16.22%)	13 (22.03%)	
Intermediate	7 (18.92%)	7 (11.86%)	
Bachelor’s degree	12 (32.43%)	18 (30.51%)	
Master’s/doctorate degree	6 (16.22%)	8 (13.56%)	
Marital status			
Married	33 (89.19 %)	4 (10.81%)	1.00
Single/divorced/widowed	53 (89.83%)	6 (10.17%)	
Children under 18 years			
Yes	23 (62.16%)	31 (52.54%)	0.70
No	9 (24.62%)	17 (28.81%)	
Current employment status			
Able to work	17 (45.95%)	28 (47.46%)	0.26
Unable to work	5 (13.51%)	15 (25.42%)	
Others (retired/student/housewife)	15 (40.54%)	16 (27.12%)	
Caregiver at home			
Spouse	23 (62.16%)	46 (77.97%)	0.10
Parents	3 (8.11%)	6 (10.17%)	
Others (kids/neighbor/sibling/self)	11 (29.73%)	7 (11.86%)	
Head of the family			
Yes	16 (43.24%)	33 (55.93%)	0.29
No	21 (56.76%)	26 (44.07%)	
SES			
Low SES	2 (5.41%)	11 (18.64%)	0.07
Middle SES	30 (81.08%)	36 (61.02%)	
High SES	5 (13.51%)	12 (20.34%)	
Treatment cost management			
Self-support	21 (56.76%)	31 (52.54%)	0.19
Family/relative support	7 (18.92%)	7 (11.86%)	
Welfare from treating hospital	5 (13.51%)	18 (30.51%)	
Support from community	4 (10.81%)	3 (5.08%)	
Health insurance			
Yes	6 (16.22%)	7 (11.86%)	0.55
No	31 (83.78%)	52 (88.14%)	

Patients who had undergone AC had better KPS at the time of interview than the other group (p = 0.037), although 78.38% (n = 29) of the patients who had undergone AC had a malignant disease, whereas only 57.63% (n = 34) of the patients in the CC group had a malignant disease. The total number of patients suffering from clinical depression according to the PHQ-9 Scale in the study were 41 (42.7%), of which 12 (12.5%) were in the AC group and 29 (30.2%) were in the CC group. The median PHQ-9 Scale score in the AC group was 6 (range: 3-10), which was less than the median PHQ-9 Scale score in the conventional craniotomy group, which was 9 (range: 4-12). This difference, however, was not statistically significant (p = 0.06). Table 2 includes a detailed comparison of all the clinical variables and treatment details between the two groups.

**Table 2:** Comparison of clinical variables between the two groups

Parameters	Awake craniotomy ( N = 37)	Conventional craniotomy ( N = 59)	P-value
Currently smoking			
Yes	4 (10.81%)	9 (15.25%)	0.76
No	33 (89.19%)	50 (84.75%)	
History of psychological distress prior to diagnosis of brain tumor			
Yes	1 (2.70%)	4 (6.78%)	0.29
No	36 (97.30%)	55 (93.22%)	
Karnofsky Performance Score (functional status)			
KPS score > 70	31 (83.78%)	37 (62.71%)	0.03
KPS score ≤ 70	6 (16.22%)	22 (37.29%)	
Treatment stage at the time of interview			
Only surgical procedures done	3 (8.11%)	13 (22.03%)	0.01
Referred to oncology after surgery	5 (13.51%)	13 (22.03%)	
Oncology treatment started/continued	16 (43.24%)	9 (15.25%)	
Treatment completed/follow-ups	13 (35.14%)	24 (40.68%)	
Current use of steroids			
Yes	4 (10.81%)	15 (25.42%)	0.11
No	33 (89.19%)	44 (74.58%)	
Current use of antiepileptic drugs			
Yes	17 (45.95%)	31 (52.54%)	0.67
No	20 (54.05%)	28 (47.46%)	
Tumor type			
Low grade (grade I and II)	8 (21.62%)	25 (42.37%)	0.04
High grade (grade III and IV)	29 (78.38%)	34 (57.63%)	
Hemispheric lateralization			
Left	27 (72.79%)	32 (54.24%)	0.10
Right	9 (24.32%)	26 (44.07%)	
Not specified	1 (2.70%)	1 (1.69%)	
Tumor grade			
Grade I	5 (13.51%)	7 (11.86%)	0.59
Grade II	9 (24.32%)	20 (33.90%)	
Grade III	13 (35.14%)	17 (28.81%)	
Grade IV	8 (21.62%)	8 (13.56%)	
Not specified	2 (5.41%)	7 (11.86%)	
Cognitive impairment			
Yes	2 (5.41%)	6 (10.17%)	0.48
No	35 (94.59%)	53 (89.83%)	
Tumor recurrence			
Yes	7 (18.92%)	12 (20.34%)	1.00
No	30 (81.08%)	47 (79.66%)	
Duration since diagnosis (in months)	Median: 7 (2–15)	Median: 6 (1–17)	0.52
Patient Health Questionnaire-9 (PHQ-9) Scale scores	6.72 ± 5.17 (median: 6 (3–10))	8.86 ± 8.86 (median: 9 (4–12))	0.06

## Discussion

The primary goal of this study was to determine depression in patients who underwent AC for brain tumor resection and compare it with patients who underwent CC under GA. Contrary to our hypothesis, the mean and median PHQ-9 Scale scores were less in the AC group than in the other arm. This translates to less prevalence of depression in patients who underwent AC for brain tumor resection, although the difference was not statistically significant. This finding is important since up to 40% of oncology patients suffer from depression, irrespective of the treatment offered [4]. Most earlier studies on AC have studied patients’ satisfaction and acceptance of undergoing AC, and very few have assessed depression.

In one of the earlier studies, Wrede et al. compared 48 patients undergoing AC with 43 patients undergoing CC for patient acceptance of the procedure using a standardized questionnaire (PPP33) [14]. It was the first study that had employed a standard tool and reported better acceptance for AC as compared with the CC group [14]. Joswig et al. retrospectively reviewed 24 AC patients and reported similar findings [15]. van Ark et al. conducted an observational study on anxiety, memory, and coping in patients who had undergone brain tumor resection using a self-designed questionnaire [16]. Their study included 272 patients, of which 27 (9.9%) had undergone AC and the rest had undergone CC [16]. There was no gender disparity in their data. Patients who underwent AC experienced less preoperative stress and anxiety than those who underwent CC. Male patients mentioned urinary catheterization as the worst memory from the whole procedure, while female patients mentioned postoperative surgical site pain to be the worst memory. There was no significant difference in overall patient satisfaction between the two groups [16]. All these findings correlate with our data.

We have found no significant difference in depression in patients with regard to the degree of histological grade of the tumor. This is consistent with the findings of van Ark et al., who concluded that the grade of tumor was not a predictor of a higher chance of anxiety and stress in patients undergoing AC [16]. In a recently published cohort study, Rahmani et al. assessed depression and anxiety in 28 patients who underwent AC for glioma resection [17]. They used the Hospital Anxiety and Depression Scale (HADS) before and after AC. In their series, 50% of the patients with high-grade gliomas and 25 patients with low-grade gliomas reported anxiety and depression (p = 0.017) [17]. In an extensive review article, Milian et al. reported that up to 14% of the patients recalled experiencing strong nervousness and uneasiness during AC [18]. However, the vast majority felt satisfied with the anesthesia technique and mentioned that they would undergo AC again if needed because of the favorable experience. None of the studies in that review had reported the occurrence of post-traumatic stress disorder in any of their patients [18].

Studies have also been conducted on interventions to reduce intraoperative stress during AC. Wu et al. conducted a randomized study on the effects of intraoperative music listening on reducing stress and anxiety during surgery [19]. They had two groups of 19 patients each, who underwent AC. The patients included in the study group listened to music in the preoperative area and during surgery. The authors reported that listening to music significantly decreased patient stress and anxiety during AC [19]. Although this aspect was not assessed in our study, music has been shown to significantly reduce stress in different awake procedures [20,21]. Other limitations of our study are a smaller sample in the AC arm, which prevented us from performing regression analysis, and variable time since the onset of surgical treatment in different patients. Some patients were undergoing chemotherapy and/or radiotherapy, which can also have a negative impact on mental health, but we did not look into that aspect independently. We feel that despite limitations, our data is a useful addition to the extremely limited literature available on this topic from low-middle income countries, and the inclusion of a comparison group further adds strength to our study.

## Conclusions

AC for brain tumor resection is not associated with any additional occurrence of depression when compared with tumor resection under general anesthesia. Supported by the available literature, we can conclude that patients who undergo AC feel more satisfied with their surgical treatment, which is an important inference considering the remarkably high rate of anxiety and depression in patients who are diagnosed with a brain tumor.

## References

[1] Mainio A, Hakko H, Niemelä A, Koivukangas J, Räsänen P (2005). Depression and functional outcome in patients with brain tumors: a population-based 1-year follow-up study. J Neurosurg.

[2] Rooney AG, Carson A, Grant R (2011). Depression in cerebral glioma patients: a systematic review of observational studies. J Natl Cancer Inst.

[3] Starkweather AR, Sherwood P, Lyon DE, McCain NL, Bovbjerg DH, Broaddus WC (2011). A biobehavioral perspective on depressive symptoms in patients with cerebral astrocytoma. J Neurosci Nurs.

[4] Pidani AS, Rao AM, Shamim MS (2018). Depression in adult patients with primary brain tumours: a review of independent risk factors. JPMA.

[5] Szelényi A, Bello L, Duffau H (2010). Intraoperative electrical stimulation in awake craniotomy: methodological aspects of current practice. Neurosurg Focus.

[6] De Witt Hamer PC, Robles SG, Zwinderman AH, Duffau H, Berger MS (2012). Impact of intraoperative stimulation brain mapping on glioma surgery outcome: a meta-analysis. J Clin Oncol.

[7] Bukhari SS, Shamim MS (2020). Can awake glioma surgery be the new standard of care in developing countries?. Surg Neurol Int.

[8] Serletis D, Bernstein M (2007). Prospective study of awake craniotomy used routinely and nonselectively for supratentorial tumors. J Neurosurg.

[9] Liu R, Page M, Solheim K, Fox S, Chang SM (2009). Quality of life in adults with brain tumors: current knowledge and future directions. Neuro Oncol.

[10] Pidani AS, Siddiqui AR, Azam I, Shamim MS, Jabbar AA, Khan S (2020). Depression among adult patients with primary brain tumour: a cross-sectional study of risk factors in a low-middle-income country. BMJ Open.

[11] Ruis C, Wajer IH, Robe P, van Zandvoort M (2017). Anxiety in the preoperative phase of awake brain tumor surgery. Clin Neurol Neurosurg.

[12] Potters JW, Klimek M (2015). Awake craniotomy: improving the patient's experience. Curr Opin Anaesthesiol.

[13] Hejrati N, Spieler D, Samuel R, Regli L, Weyerbrock A, Surbeck W (2019). Conscious experience and psychological consequences of awake craniotomy. World Neurosurg.

[14] Wrede KH, Stieglitz LH, Fiferna A (2011). Patient acceptance of awake craniotomy. Clin Neurol Neurosurg.

[15] Joswig H, Bratelj D, Brunner T, Jacomet A, Hildebrandt G, Surbeck W (2016). Awake craniotomy: first-year experiences and patient perception. World Neurosurg.

[16] van Ark TJ, Klimek M, de Smalen P, Vincent AJ, Stolker RJ (2018). Anxiety, memories and coping in patients undergoing intracranial tumor surgery. Clin Neurol Neurosurg.

[17] Rahmani M, Hendi K, Ajam H (2020). Anxiety and depression after awake craniotomy for cerebral glioma. Iran J Surg.

[18] Milian M, Tatagiba M, Feigl GC (2014). Patient response to awake craniotomy - a summary overview. Acta Neurochir (Wien).

[19] Wu PY, Huang ML, Lee WP, Wang C, Shih WM (2017). Effects of music listening on anxiety and physiological responses in patients undergoing awake craniotomy. Complement Ther Med.

[20] Lin PC, Lin ML, Huang LC, Hsu HC, Lin CC (2011). Music therapy for patients receiving spine surgery. J Clin Nurs.

[21] Buffum MD, Sasso C, Sands LP, Lanier E, Yellen M, Hayes A (2006). A music intervention to reduce anxiety before vascular angiography procedures. J Vasc Nurs.

